# Tumor cells-derived CXCL17 is associated with up-regulated neutrophil to monocyte ratio and predicts poor prognosis in oral squamous cell carcinoma

**DOI:** 10.3389/fonc.2026.1591013

**Published:** 2026-05-21

**Authors:** Xingxing Zhao, Nisha Zhu, Xinyu Ye, Xiaofeng Huang, Yayi Hou, Qingang Hu, Yanhong Ni

**Affiliations:** 1 Department of Oral and Maxillofacial Surgery, Nanjing Stomatological Hospital, Affiliated Hospital of Medical School, Nanjing University, Nanjing, China; 2 The State Key Laboratory of Pharmaceutical Biotechnology, Division of Immunology, Medical School, Nanjing University, Nanjing, China; 3 Central Laboratory of Stomatology, Nanjing Stomatological Hospital, Affiliated Hospital of Medical School, Nanjing University, Nanjing, China

**Keywords:** CXCL17, oral squamous cell carcinoma, overall survival, prognosis factors, tumor microenvironment

## Abstract

CXCL17, the most recently identified member of the chemokine family, has been implicated in angiogenesis, tumorigenesis, infection, and inflammation across various tumors. However, the expression profile and functional characterization of CXCL17 in oral squamous cell carcinoma (OSCC) remain inadequately explored. Based on our previous proteomics studies of OSCC patients, we reported the elevated CXCL17 expression in tumor tissues compared to adjacent non-tumor tissues. We originally characterized the prognostic value of CXCL17 and its spatial and temporal distribution in 72 OSCC tissue specimens by immunohistochemistry staining. Elevated CXCL17 expression was correlated with advanced tumor stage, increased distant metastasis, and significantly shorter overall survival. Multivariate analysis confirmed tumor cell–derived CXCL17 as an independent prognostic factor for overall survival. Additionally, we investigated the relationship between CXCL17 and peripheral blood indicators hypothesizing that CXCL17 may influence peripheral immune cell regulation. We revealed that higher CXCL17 in tumor center was associated with reduced postoperative neutrophil, preoperative monocyte and postoperative leukocyte. Moreover, CXCL17 levels in tumor center was correlated significantly with preoperative neutrophil-to-lymphocyte ratio (NLR) and postoperative lymphocyte-to-monocyte ratio (LMR). Elevated postoperative neutrophil-to-monocyte ratio (NMR) was associated with advanced TNM stage and higher lymph node metastasis rates, and was also observed in patients with postoperative distant metastasis. In summary, these findings suggest that CXCL17 contributes to OSCC progression and poor prognosis by modulating systemic immune responses and reshaping the tumor immune microenvironment, highlighting its potential as both a prognostic biomarker and a therapeutic target.

## 1 Introduction

Oral cancer ranks among the ten most common malignancies globally and oral squamous cell carcinoma (OSCC) accounts for about 90% of oral cancer (1). Although advances in diagnostic and therapeutic techniques have led to a yearly decline in overall cancer incidence and mortality rates, the incidence of oral cancer has been increasing by 1% annually (2). Unfortunately, OSCC have a poor prognosis, with limited improvements in survival over the past several decades (3, 4). The prognosis of OSCC was typically correlated with tumor staging and grading. Cancer invasion and metastasis are recognized as the result of complex interactions among various cell types within the tumor microenvironment (5). Increasing evidence suggests that chemokines serve not only as leukocyte chemoattractant but also exert pleiotropic effects on immune modulation, cellular proliferation, tumor growth, and cancer cell invasion and homing, all of which are crucial for metastasis (6).

CXC ligand 17 (CXCL17) is a novel 119-amino acid CXC chemokine, known as the most recent member of the CXCL family (7). Initially discovered in 2006, it was referred to as vascular endothelial growth factor-related chemokine 1 (VCC-1) (8), dendritic cell and monocyte chemokine-like protein (DMC) (9). Recent research has highlighted that CXCL17 is a chemokine that is restricted to mucosal expression. Quantification of the average level of CXCL17 expression in various organs revealed that the highest expression was in the trachea and bronchus, but also in the oral mucosa, tongue, esophagus etc. (10). Under pathological conditions, the expression level of CXCL17 in these organs is often significantly increased (11). Matsui et al. demonstrated that NIH3T3 cells transfected with CXCL17 formed tumors more rapidly than cells, with elevated blood flow signals observed in the experimental group compared to control group (12). CXCL17 is associated with vascular endothelial growth factor (VEGF) and may promote tumor growth in solid tumors by enhancing angiogenesis (8). Additionally, CXCL17 has been identified as a direct target of miR-4513; knocking down the CXCL17 expression could inhibit the effects of miR-4513 on proliferation and apoptosis of OSCC cells (13). However, other studies suggest that CXCL17 may play a role in anti-tumor immune responses during pancreatic carcinogenesis. In premalignant intraductal papillary mucinous neoplasm, CXCL17 induces dendritic cells accumulation at the tumor site, enhancing tumor cell susceptibility to cytotoxic T cell-mediated cytolysis (14). Despite these insights, the clinical role of CXCL17 in OSCC remains to be fully elucidated.

Inflammation plays a crucial role in carcinogenesis by promoting primary tumor growth, stimulating tumor cell proliferation, inhibiting apoptosis, and increasing mitotic rates (15). And examples of inflammatory conditions that favor the development of cancer can be found in many organs including ovary (16), pancreas (17), esophagus (18), stomach (19), liver (20), bladder (21), colon (22), lung (23) and endometrium (24). For instance, the inflammation-induced expression of CCL11 in both colonic epithelial and lamina propria immune cells serves as a key mechanism which exacerbates colitis and promotes inflammation-associated colon tumorigenesis (25). Among various parameters of systemic inflammatory response, the neutrophil-to-lymphocyte ratio (NLR) and lymphocyte-to monocyte ratio (LMR) have emerged as significant prognostic indicators in several malignancies. In colorectal cancer, patients with higher NLR but lower LMR were significantly associated with decreased overall survival and disease-free survival (26). In advanced pancreatic cancer, both NLR and LMR were independent prognostic factors, unaffected by other clinical variables (27, 28). Additionally, the neutrophil to monocyte ratio (NMR) has been suggested as a potential index for identifying disease or predicting prognosis in skin cancer (29) and breast cancer (30). In colon cancer, tumor cell-secreted CXCL17 has been shown to promote tumor growth via the recruitment of myeloid-derived CD11b^+^Gr1^high^F4/80^−^ granulocytic cells to tumor site, thereby increasing the number of CD31^+^ vascular structures (31). This suggests that CXCL17 may preferentially recruit cells with granulocytic nature. Therefore, we hypothesize that elevated CXCL17 expression could alter the subsets of lymphocytes, monocytes, and neutrophils, potentially impairing the immune system’s defense against malignant tumors (32).

Interestingly, our previous proteomic studies indicated that the CXCL17 levels were remarkably elevated in tumor tissues compared to normal tissues but the clinical significance of CXCL17 in the development of OSCC remains unclear. We evaluated the correlation between CXCL17 expression and clinicopathological parameters and then considered that tumor infiltrating CXCL17+ cells could serve as an independent prognostic factor for the survival rate of OSCC patients. Given that local and systemic inflammation play very critical roles in the apparition and development of tumors and metastasis (33), we further investigated the interrelation of CXCL17 and NLR, LMR, NMR in peripheral blood of OSCC patients. Our findings revealed that CXCL17 was relevant to systemic inflammation in OSCC.

## 2 Materials and methods

### 2.1 Patients and tissue specimens

We utilized archived formalin-fixed, paraffin-embedded tissues from 72 patients with primary OSCC who underwent initial curative surgical resection at the Department of Pathology, Nanjing Stomatological Hospital, between 2006 and 2010. Ethical approval for the use of these OSCC specimens was obtained from the Research Ethics Committee of Nanjing Stomatology Hospital. All patients were followed from the date of surgery until March 1, 2016, death, or loss to follow-up, whichever occurred first. Inclusion criteria were as follows (1): patients underwent initial curative surgical resection for primary OSCC; (2) no preoperative non-surgical treatments (radiotherapy, chemotherapy, biotherapy, or other non-surgical modalities); (3) no systemic comorbidities such as hypertension or diabetes mellitus; not pregnant at the time of diagnosis and treatment; (4) intraoperative frozen section confirmed negative surgical margins. Exclusion criteria were as follows: (1) distant metastasis or unresectable disease; (2) history of other malignancies; (3) severe dysfunction of vital organs (heart, lung, liver, kidney, etc.); (4) coagulation disorders or bleeding tendency; (5) immune system disorders or long-term use of immunosuppressants; (6) psychiatric disorders or cognitive impairment that would preclude treatment compliance or follow-up; (7) positive surgical margins on intraoperative frozen section; (8) refusal to provide informed consent or poor treatment adherence. Curative resection for OSCC was defined as complete resection of all tumor nodules, with a resection margin of at least 5 mm, ensuring that the cut surface was free of tumor based on histological examination. All OSCC tissues were evaluated according to World Health Organization (WHO) classifications by two independent pathologists.

### 2.2 Protein extraction and sample preparation for mass spectrometry

The tissues were then lysed using EasyPep Lysis buffer. In accordance with the manufacturer’s suggested technique, the protein extraction concentration was further modified for the subsequent sample digestion and purification using the EasyPep MS sample preparation kit (#A40006, ThermoFisher, USA). In summary, proteins were concurrently reduced and alkylated using a reduction and alkylation solution, and they were then incubated for ten minutes at 95 °C. Trypsin and Lys-C were used to digest fifty micrograms of proteins from each sample for an hour at 37 °C. Wash solutions and a reverse phase-based C18 spin column were used to clean up the digested peptides. After drying, 0.1% formic acid (FA) was used to reconstitute the purified peptides. Before mass spectrometric analysis, peptides were measured using a peptide assay (#23275, ThermoFisher, USA) and diluted to a concentration of 0.25 µg/µL with an equal loading amount of 1 µg injection.

### 2.3 Liquid chromatography-mass spectrometry

One microgram of the purified peptide samples was analyzed by Nano LC-coupled Trapped Ion Mobility Spectrometry Time-of-Flight Pro2 Mass Spectrometer (TimsTOF; Bruker, USA). For the sample fractionation in LC-MS, solvent A (0.1% FA in H2O) and solvent B (0.1% FA in acetonitrile) were used as the gradient. Optimized MS settings were applied to maximize the performance on protein identifications.

For TimsTOF, peptides were loaded onto the nanoElute 2 HPLC by loading buffer at 0.3 μL/min at 50°C and then separated on an Aurora Ultimate CSI Column (120 Å, 25 cm × 75 µm, 1.7 µm, C18, Ionopticks, AU) using the Dionex UltiMate 3000 RSLCnano NanoLC system connected to the TimsTOF Pro2. For Data-Independent Acquisition with Parallel Accumulation Serial Fragmentation (DIA-PASEF) experiments, the range of MS1 scan was 100–1,700 m/z in positive mode. 25 Da m/z isolation windows were assigned over the mass range of 400–1,000 m/z with a 100% duty cycle, 100 ms accumulation, and ramp time in the TIMS.

### 2.4 Proteomic analysis

The raw data files generated from timsTOF were with the help of Novel Bioinformatics Co., Ltd (Shanghai, China).

### 2.5 Detection of peripheral blood indicators

For complete blood count analysis using the fully automated hematology analyzer (Sysmex Corporation, XS-900i, Japan), venous blood (2 mL) is collected in EDTA-K_2_ tubes, gently mixed, and analyzed within 2 hours. After self-check, the instrument automatically aspirates and dilutes the sample (1:200), then analyzes it via two channels: the electrical impedance channel counts red blood cells, white blood cells, and platelets based on the Coulter principle, while the optical scattering channel performs a five-part white blood cell differential using laser-based size and complexity analysis. The neutrophil-to-lymphocyte ratio (NLR) was calculated by dividing the absolute count of neutrophils by the absolute count of lymphocytes. The lymphocyte-to-monocyte ratio (LMR) was defined as the ratio of the absolute lymphocyte count to the absolute monocyte count. The neutrophil-to-monocyte ratio (NMR) was calculated by dividing the absolute count of neutrophils by the absolute count of monocytes.

### 2.6 Immunohistochemistry

Paraffin sections (4 μm thick) were deparaffinized and rehydrated in an ethanol series. Heat-mediated antigen retrieval was conducted in 10 mmol citric acid solution (pH 6.0), followed by blocking of endogenous peroxidase activity with a 3% hydrogen peroxide solution. After washing in PBS (pH 7.4) for three times, the slides were incubated overnight at 4 °C with primary antibodies directed against CXCL17 (#MAB42071, Mouse anti-human, R&D Systems, Minneapolis, USA) with dilutions of 1:2000 in PBS containing 5% bovine serum albumin. After washed in PBS three times, sections were incubated in peroxidase-labeled anti-mouse antibodies (#K4001, Rabbit anti-mouse, DAKO, Carpinteria, CA, USA) at 37 °C for 3*0 minutes.* Finally, all section performed the DAB (#P0203, Horseradish Peroxidase Color Development Kit, Beyotime Biotechnology, China) chromogen and nuclear staining.

### 2.7 Quantification of CXCL17 expression

All IHC slides were captured with Hamamatsu scanner (NanoZoomer‐XR C12000, Japan) and calculated using viewing software (NDP.view2, U12388‐01, HAMAMATSU). First of all, we defined the tumor center and tumor invasive front regions in each OSCC specimens by recognizing classic squamous cell morphology. And then, each sample was counted four indicators recorded as follows: CXCL17 expression in tumor center (TC), tumor stroma (TS), tumor invasive front (TIF) and stroma of invasive front (SIF). Two experienced pathologists who were blinded to the clinicopathological information are responsible for the quantification. CXCL17-secreting cells were positively stained greyish or dark brown in cytoplasm. In each region, we calculated the percentage of positive cells in 3 randomly selected high power field (×400) and then take the average as the final immunohistochemical scores. Accordingly, the immunoreactivity score of each region was categorized into high and low expression subgroups based on the median value.

### 2.8 Data analysis

Immunostaining parameters were categorized into subgroups based on their median values. Correlations between immunostaining parameters and clinicopathological features were analyzed using chi-square tests or Fisher’s exact test, as appropriate. Survival analysis was conducted using the Kaplan-Meier method, with comparisons made using the log-rank test. A multivariate Cox proportional hazards regression model was employed to estimate the adjusted hazard ratio (HR) and 95% confidence interval (CI) and to identify independent prognostic factors. To compare clinicopathological parameters among patients, Mann-Whitney U test, Pearson’s chi-square test, Fisher’s exact test or Cochran-Mantel-Haenszel χ2 test were utilized. Kaplan–Meier analysis was used to plot the survival curve. Log-rank test was used to compare patient survival between subgroups. All experimental data presented in the figures are expressed as the mean± standard deviation from at least three independent experiments. Statistical significance was determined using ANOVA. All statistical analyses were performed with SPSS 17.0. Significance was accepted at *P*<0.05 unless otherwise noted.

## 3 Results

### 3.1 CXCL17 expression was significantly higher in OSCC tissues compared to paired adjacent none tumor tissues

**Figure 1:**
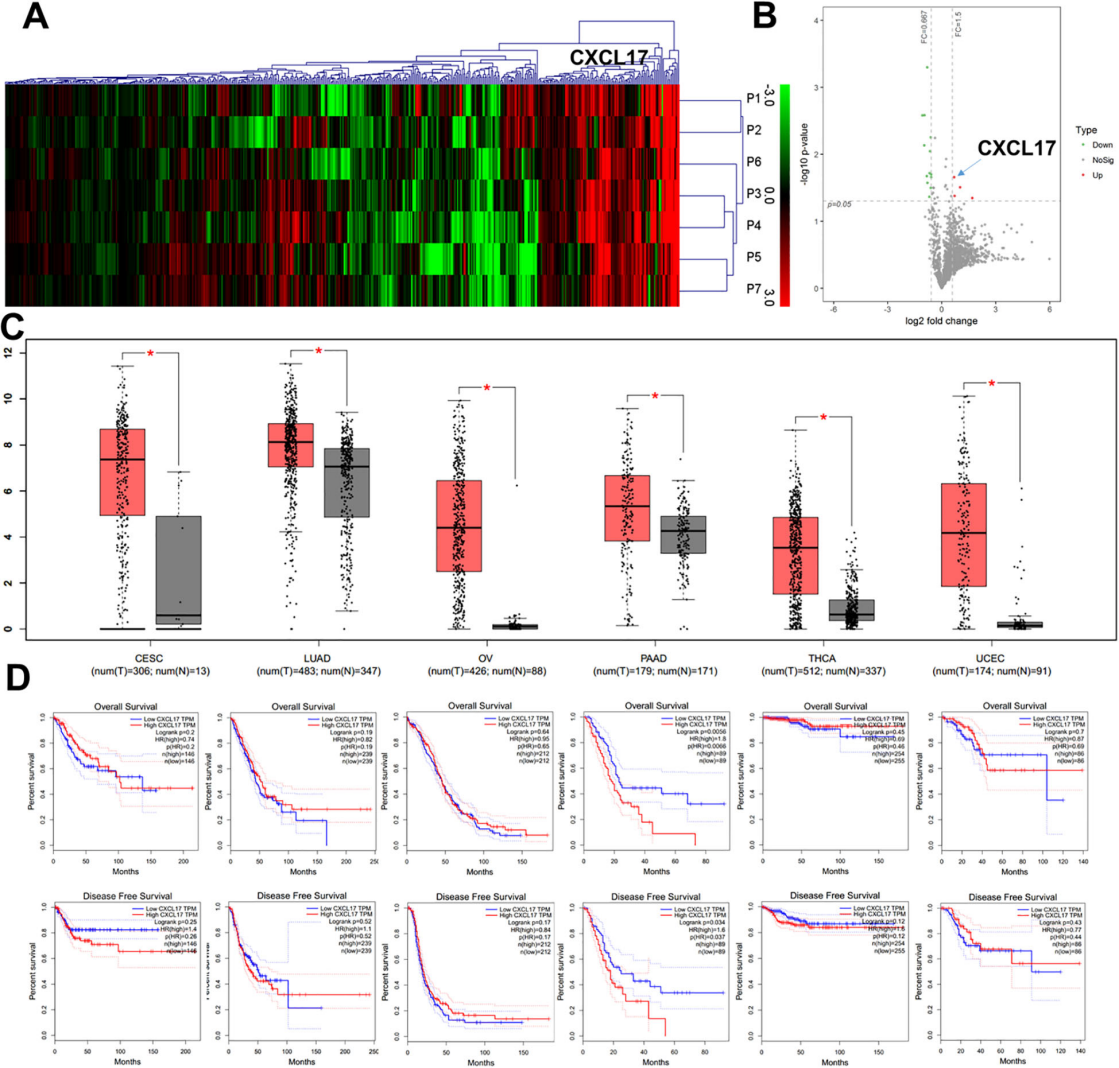
Elevated expression of CXCL17 in OSCC tissues. **(A, B)** The proteomics studies and bioinformatics analysis in seven paired tumor specimens and adjacent non-tumor tissues. **(C, D)** RNA expression of CXCL17 was significantly abundant and closely related to survival rates in various cancers through GEPIA analysis based on TCGA and GTEx data.

We collected tumor specimens and paired adjacent non-tumor tissues from seven OSCC patients for proteomics studies which were performed in Novel Bioinformatics Co., Ltd (Shanghai, China) (34). The proteomics results revealed a higher expression of CXCL17 in tumor tissues compared to adjacent non-tumor tissues (**Figures 1A, B**). To validate the results from proteomics, we utilized GEPIA (Gene Expression Profiling Interactive Analysis), a web-based tool for data mining and deeper understanding of CXCL17 gene functions based on TCGA and GTEx data. As it is shown, RNA expression of CXCL17 was significantly more abundant in various cancers compared to normal tissues, particularly in cervical squamous cell carcinoma and endocervical adenocarcinoma (CESE), lung adenocarcinoma (LUAD), ovarian serous cystadenocarcinoma(OV), pancreatic adenocarcinoma (PAAD), thyroid carcinoma (THCA), uterine corpus endometrial carcinoma(UCEC) (**Figure 1C**). Moreover, high CXCL17 expression was associated with an observably decreased overall survival (*P* = 0.0056) and disease-free survival (*P* = 0.034) in pancreatic adenocarcinoma; however, no significant impact on overall survival or disease-free survival was observed in other cancer types (**Figure 1D**).

### 3.2 CXCL17 expression increases from dysplasia to OSCC

**Figure 2:**
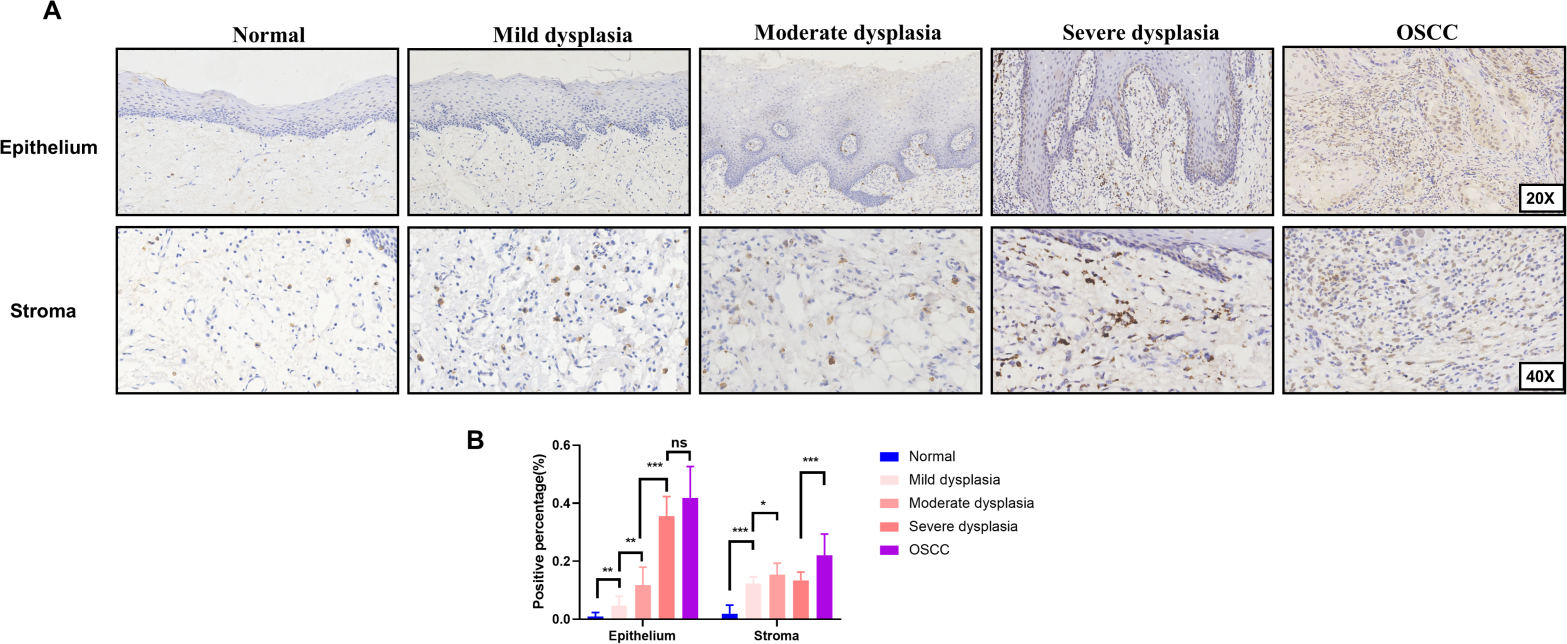
The temporal distribution of CXCL17 during the development of OSCC. **(A)** Immunohistochemistry in normal epithelial mucosa and different grades of dysplasia tissues including mild, moderate and severe dysplasia compared with OSCC tissues in epithelial cells(1st row) and stromal cells(2nd row), respectively. **(B)** Quantitative analysis of immunohistochemistry. * p < 0.05, ** p < 0.01, *** p < 0.001, statistically significant. ns, p > 0.05, not significant.

We evaluated the temporal distribution of CXCL17 during the development of OSCC through immunohistochemical analysis. CXCL17 were primarily localized in the cytoplasm of tumor cells, with occasional presence in the cytoplasm or nuclei of stromal cells. The expression patterns of CXCL17 in epithelial cells and stroma cells were assessed across normal oral mucosa, mild dysplasia, moderate dysplasia, severe dysplasia, and OSCC *in situ* respectively. Consistent with our proteomics findings, CXCL17 expression was much higher in both tumor cells and tumor stroma than in normal epithelium and subepithelial stroma (**Figure 2A**). Additionally, CXCL17 expression was up-regulated during the malignant progression of OSCC in the epithelial area, indicating its role in the carcinomatous changes of epithelial cells. Notably, the infiltration of CXCL17^+^ stromal cells also increased from dysplasia to tumor when compared to normal tissues (**Figure 2B**).

### 3.3 The spatial distribution of CXCL17 in OSCC

**Figure 3:**
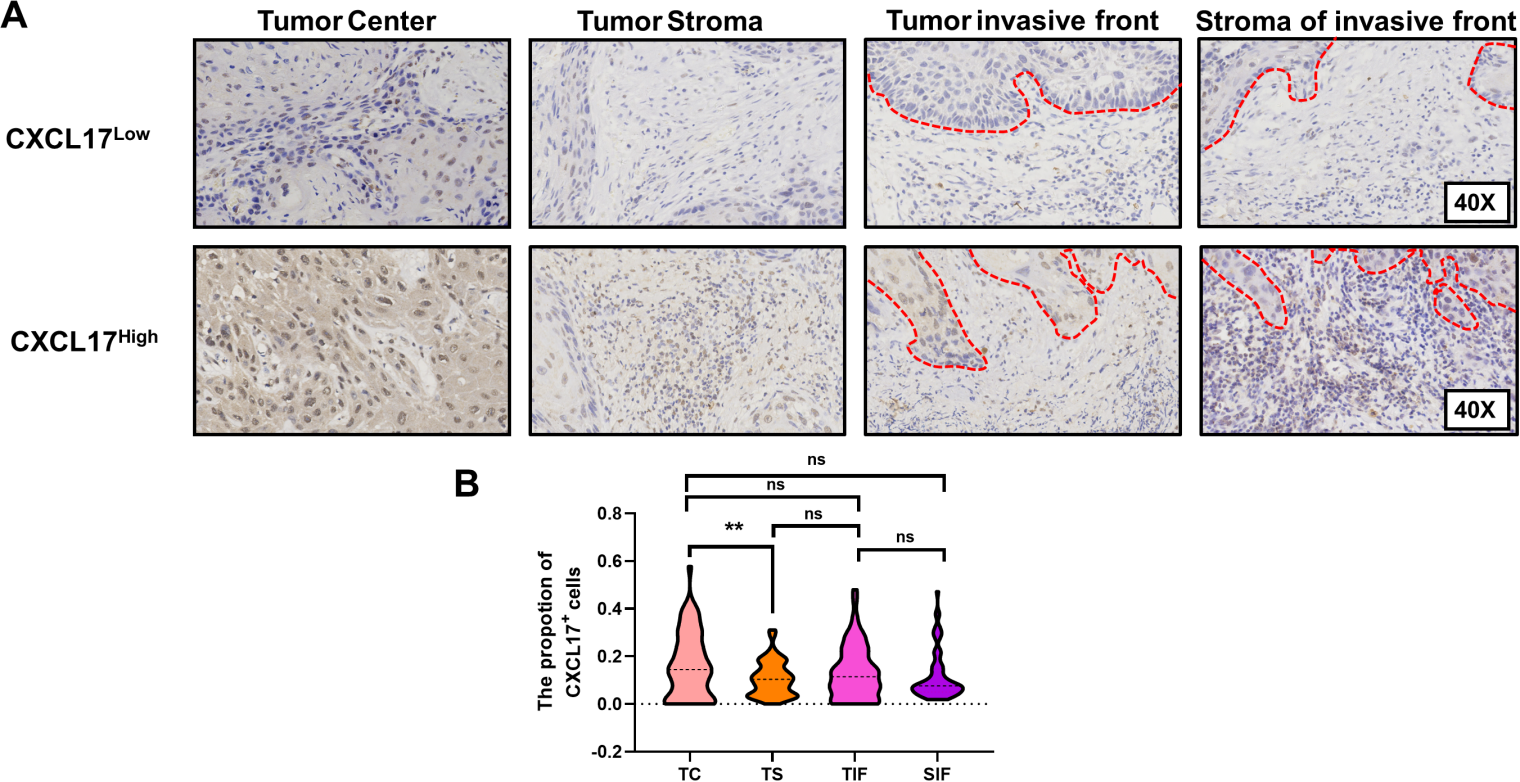
The spatial distribution of CXCL17 in OSCC. **(A)** Immunohistochemistry of CXCL17 expression in different regions including TC, tumor center; TS, tumor stroma; TIF, tumor invasive front and SIF, stroma of invasive front. **(B)** Quantitative analysis of CXCL17 among different regions. ** p < 0.01, statistically significant. ns, p > 0.05, not significant.

The regional heterogeneity of the tumor microenvironment prompted us to investigate the spatial distribution of CXCL17 in tumor center (TC), tumor stroma(TS), tumor invasive front (TIF) and stroma of invasive front (SIF), respectively (**Figure 3A**). Nonparametric tests were employed to compare the rates of positively expressed cells across these distinct regions. In high-power fields, the mean proportion of CXCL17^+^ cells was as follows: TC: 0.177 ± 0.158, TS: 0.120 ± 0.093, TIF: 0.166 ± 0.182, and SIF: 0.116 ± 0.095. The results indicated that CXCL17 expression was much higher in TC compared to TS (*P* = 0.0031). However, there was no significance between TC and TIF (*P* = 0.189), or TS and SIF (*P* = 0.556) (**Figure 3B**).

### 3.4 Correlations between CXCL17 expression and clinicopathological characteristics in OSCC patients

To evaluate the correlation between CXCL17 expression and tumor development, we also analyzed the association between CXCL17 expression and the clinicopathological characteristics of 72 primary OSCC patients. Specifically, we examined the correlations between the CXCL17^+^ cells in distinct regions and various clinicopathologic characteristics including patient age, gender, smoking habit, lymph node metastasis status, tumor stage (TNM), predominant pattern of invasion (PPOI), worst pattern of invasion (WPOI), tumor differentiation, along with recurrence and metastasis after surgery (**Table 1**). Chi‐square and Fisher’s exact tests revealed that high levels of CXCL17^+^ tumor cells in TC and TIF was significantly associated with postoperative metastasis in OSCC patients (*P* = 0.005 and 0.047, respectively). A strong correlations was also found between CXCL17 in TS and postoperative metastasis (*P* = 0.005), while no significant association was observed between CXCL17 expression in SIF and postoperative metastasis (*P* = 0.233). Moreover, CXCL17^+^ stromal cells within the tumor nest showed a significant relevance to tumor TNM stage of OSCC patients (*P* = 0.009). However, other clinicopathological characteristics such age, gender, smoking habit, lymph node metastasis status, PPOI, WPOI, tumor differentiation and recurrence, were not statistically associated with CXCL17 expression in any region.

**Table 1:** Association between CXCL17 expression and clinicopathological parameters in OSCC patients.

Variables	n= (72)	CXCL17 in TC		χ²	*P*	CXCL17 in TS		χ²	*P*	CXCL17 in TIF		χ²	*P*	CXCL17 in SIF		χ²	*P*
		Low	High			Low	High			Low	High			Low	High		
Sex																	
Male	36	14(38.9)	22(61.1)	3.556	0.059	15(41.7)	21(58.3)	2	0.157	18(50.0)	18(50.0)	0.000	1.000	17(47.2)	19(52.8)	0.222	0.637
Female	36	22(61.1)	14(38.9)			21(58.3)	15(41.7)			18(50.0)	18(50.0)			19(52.8)	17(47.2)		
Age(years)																	
<60	49	24(49.0)	25(51.0)	0.064	0.8	27(55.1)	22(44.9)	1.597	0.206	25(51.0)	24(49.0)	0.064	0.800	24(49.0)	25(51.0)	0.064	0.8
≥60	23	12(52.2)	11(47.8)			19(82.6)	4(17.4)			11(47.8)	12(52.2)			12(52.2)	11(47.8)		
Smoking																	
No	58	27(46.6)	31(53.4)	2.185	0.139	28(48.3)	30(51.7)	0.747	0.387	29(50.0)	29(50.0)	0.063	0.802	29(50.0)	29(50.0)	0.063	0.802
Yes	13	9(69.2)	4(30.8)			8(61.5)	5(38.5)			7(53.8)	6(46.2)			7(53.8)	6(46.2)		
TNM																	
I-II	35	18(51.4)	17(48.6)	0.056	0.814	23(65.7)	12(34.3)	6.727	0.009**	19(54.3)	16(45.7)	0.500	0.479	20(57.1)	15(42.9)	1.390	0.238
III-IV	37	18(58.6)	19(51.4)			13(35.1)	24(64.9)			17(45.9)	20(54.1)			16(43.2)	21(56.8)		
PPOI																	
1-3	60	30(50.0)	30(50.0)	0	1	31(51.7)	29(48.3)	0.4	0.527	29(48.3)	31(51.7)	0.400	0.527	29(48.3)	31(51.7)	0.400	0.527
4-5	12	6(50.0)	6(50.0)			5(41.7)	7(58.3)			7(58.3)	5(41.7)			7(58.3)	5(41.7)		
WPOI																	
1-3	49	26(53.1)	23(46.9)	0.575	0.448	27(55.1)	22(44.9)	1.597	0.216	26(53.1)	23(46.9)	0.575	0.448	28(57.1)	21(42.9)	3.130	0.077
4-5	23	10(43.5)	13(56.5)			9(39.1)	14(60.9)			10(43.5)	13(56.5)			8(34.8)	15(65.2)		
Differentiation																	
Well	26	13(50.0)	13(50.0)	0	1	12(46.2)	14(53.8)	0.241	0.624	14(46.2)	12(53.8)	0.241	0.624	13(50.0)	13(50.0)	0.000	1.000
Moderate to poor	46	23(50.0)	23(50.0)			24(52.2)	22(47.8)			22(47.8)	24(52.2)			23(50.0)	23(50.0)		
Lymphatic metastasis																	
No	44	23(52.3)	21(47.7)	0.234	0.629	25(56.8)	19(43.2)	2.104	0.147	24(54.5)	20(45.5)	0.935	0.334	24(54.5)	20(45.5)	0.935	0.334
Yes	28	13(46.4)	15(53.6)			11(39.3)	17(60.7)			12(42.9)	16(57.1)			12(42.9)	16(57.1)		
Metastasis																	
No	65	36(55.4)	29(44.6)	7.754	0.005**	36(55.4)	29(44.6)	7.754	0.005**	35(53.8)	30(46.2)	3.956	0.047*	34(52.3)	31(47.7)	1.424	0.233
Yes	7	0(0.0)	7(100.0)			0(0.0)	7(100.0)			1(14.3)	6(85.7)			2(28.6)	5(71.4)		
Recurrence																	
No	63	31(49.2)	32(50.8)	0.127	0.722	31(49.2)	32(50.8)	0.127	0.722	30(47.6)	33(52.4)	1.143	0.285	29(46.0)	34(54.0)	3.175	0.075
Yes	9	5(55.6)	4(44.4)			5(55.6)	4(44.4)			6(66.7)	3(33.3)			7(77.8)	2(22.2)		
Total	72	36	36														

*P < 0.05, **P < 0.01, statistically significant. TC, tumor center; TS, tumor stroma; TIF, tumor invasive front; SIF, stroma of invasive front; WPOI, worst pattern of invasion; PPOI, primary pattern of invasion.

Bold values indicate statistical significance. The corresponding P values are shown in the table.

### 3.5 High CXCL17 expression predicts poor prognosis in OSCC patients

**Figure 4:**
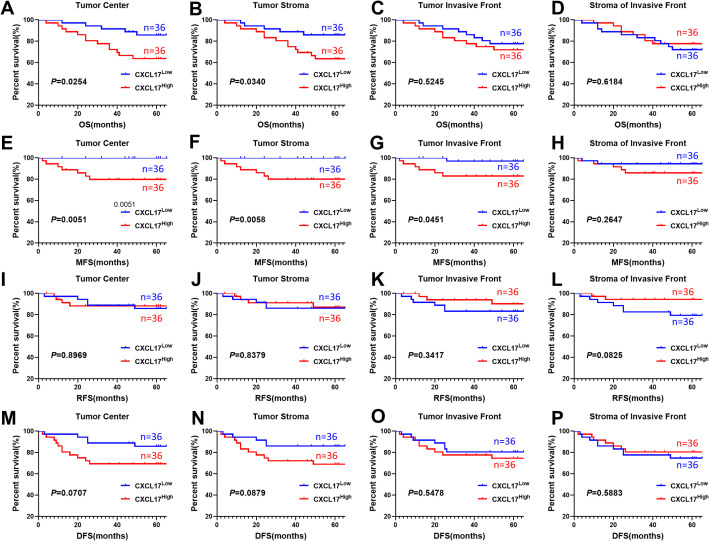
Kaplan–Meier survival curves of correlations between CXCL17 and prognosis of OSCC patients in different regions. **(A–D)** Overall survival analysis in TC, TS, TIF and SIF. **(E–H)** Recurrence-free survival analysis. **(I–L)** Metastasis-free survival analysis. **(M–P)** Disease-free survival analysis.

To assess whether CXCL17 could serve as a prognostic marker for OSCC, we plotted Kaplan-Meier survival curves to examine the correlation between CXCL17 expression in distinct regions and survival rates, including overall survival, recurrence-free survival, metastasis-free survival and disease-free survival (**Figure 4**). Follow-up was completed on January 8, 2019, with a median follow-up duration of 60 months (range, 12–127 months). The CXCL17 high and CXCL17 low subgroups were categorized based on the median proportion of CXCL17^+^ cells per section. The median value of each group was 0.149 in TC, 0.105 in TS, 0.115 in TIF and 0.076 in SIF, respectively. Statistical analysis revealed that higher proportion of CXCL17^+^ tumor cells in TC was significantly correlated with worse overall survival (*P* = 0.0254, **Figure 4A**), metastasis-free survival (*P* = 0.0051, **Figure 4E**), whereas no significant associations were found for recurrence-free survival (*P* = 0.897, **Figure 4I**) or disease-free survival (*P* = 0.0707, **Figure 4M**). Additionally, patients with higher proportion of CXCL17^+^ stromal cells in TS tended to have shorter overall survival (*P* = 0.034, **Figure 4B**) and metastasis-free survival (*P* = 0.0058, **Figure 4F**), while no significant impact on recurrence-free survival (*P* = 0.838, **Figure 4J**) or disease-free survival (*P* = 0.0879, **Figure 4N**). Interestingly, CXCL17^+^ tumor cells in TIF compartments showed slight influence on metastasis-free survival (*P* = 0.0451, **Figure 4G**) while no significant effects were observed on overall survival, recurrence-free survival or disease-free survival (**Figure 4C**, P = 0.525; **Figure 4K**, P = 0.342; **Figure 4O**, P = 0.548, respectively). However, CXCL17+ stromal cells in SIF compartments did not demonstrate any significant influence on overall survival (**Figure 4D**), metastasis-free survival (**Figure 4H**), recurrence-free survival (**Figure 4L**), or disease-free survival(**Figure 4P**) (all *P* > 0.05, data not shown).

### 3.6 CXCL17 in TC is an independent prognostic factor for OSCC

As shown in **Tables 2**, **3**, the prognostic value of each clinicopathologic feature was ultimately identified by univariate and multivariate analysis. Univariate analyses indicated that gender, age, smoking habit, differentiation, PPOI, WPOI, lymph node metastasis status along with CXCL17 in TIF and SIF were not significantly associated with overall survival (*P*>0.05). Nevertheless, tumor stage, CXCL17 in TC and TS were correlated with overall survival (*P* = 0.044, 0.034, 0.044, respectively). Importantly, multivariate analyses indicated that CXCL17 expression in TC was an independent prognostic factor for overall survival in OSCC patients (*P* = 0.016). Additionally, lymph node metastasis status in OSCC was significantly associated with metastasis-free survival (*P* = 0.027), while CXCL17 expression showed no relevance to metastasis-free survival in either univariate or multivariate analyses. Therefore, our findings suggest that tumor cell‐derived CXCL17 in the TC regions could serve as an independent prognostic factor for OSCC, positioning it as a potential clinical tool for risk stratification. This could aid in identifying high-risk patients who may benefit from more aggressive or tailored treatment strategies.

**Table 2:** Cox-regression analyses of overall survival for clinicopathological parameters in OSCC patients.

Variables	Univariate analysis		*P*	Multivariate analysis		*P*
	HR	95% CI		HR	95% CI	
Gender						
Male versus female	0.926	0.368-2.334	0.871			
Age						
<60 versus >=60	1.731	0.683-4.387	0.248			
TNM						
I-II versus III-IV	2.887	1.029-8.103	0.044*	1.285	0.261-6.337	0.758
Smoke						
No versus Yes	0.892	0.256-3.105	0.858			
Differentiation						
Well versus Moderate to poor	1.55	0.553-4.349	0.405			
Lymphatic metastasis						
No versus Yes	2.302	0.908-5.836	0.079			
**PPOI**	0.253	0.034-1.899	0.181			
I–IIIversus IV–V						
WPOI						
I–IIIversus IV–V	0.391	0.113-1.351	0.138			
CXCL17 in TC						
Low versus High	3.048	1.086-8.560	0.034*	4.954	1.343-18.270	0.016*
CXCL17 in TS						
Low versus High	2.89	1.030-8.110	0.044*	2.077	0.547-7.894	0.283
CXCL17 in TIF						
Low versus High	1.349	0.532-0.419	0.528			
CXCL17 in SIF						
Low versus High	0.791	0.312-2.004	0.621			

*P < 0.05, statistically significant. HR, Hazard Ratio; 95CI, 95% Confidence Interval; TC, tumor center; TS, tumor stroma; TIF, tumor invasive front; SIF, stroma of invasive front; PPOI, primary pattern of invasion; WPOI, worst pattern of invasion.

Bold values indicate statistical significance. The corresponding P values are shown in the table.

**Table 3:** Cox-regression analyses of Metastasis-free survival for clinicopathological parameters in OSCC patients.

Variables	Univariate analysis		*P*	Multivariate analysis		*P*
	HR	95% CI		HR	95% CI	
Gender						
Male versus female	0.385	0.075-1.893	0.254			
Age						
<60 versus >=60	1.541	0.345-6.888	0.571			
TNM						
I-II versus III-IV	6.522	0.784-54.267	0.083			
Smoke						
No versus Yes	0.737	0.089-6.124	0.778			
Differentiation						
Well versus Moderate to poor	3.482	0.419-28.927	0.248			
Lymphatic metastasis						
No versus Yes	10.835	1.303-90.108	0.027*	7.301	0.415-128.425	0.174
PPOI						
I–IIIversus IV–V	0.037	0.000-154.988	0.438			
WPOI						
I–IIIversus IV–V	0.345	0.042-2.868	0.325			
CXCL17 in TC						
Low versus High	73.72	0.166-32760.893	0.167			
CXCL17 in TS						
Low versus High	71.374	0.162-31439.946	0.169			
CXCL17 in TIF						
Low versus High	6.534	0.786-54.292	0.082			
CXCL17 in SIF						
Low versus High	2.465	0.478-12.711	0.281			

*P < 0.05, statistically significant. HR, Hazard Ratio; 95CI, 95% Confidence Interval. TC, tumor center; TS, tumor stroma; TIF, tumor invasive front; SIF, stroma of invasive front; PPOI, primary pattern of invasion; WPOI, worst pattern of invasion.

### 3.7 CXCL17 was relevant to systemic inflammation of OSCC

**Figure 5:**
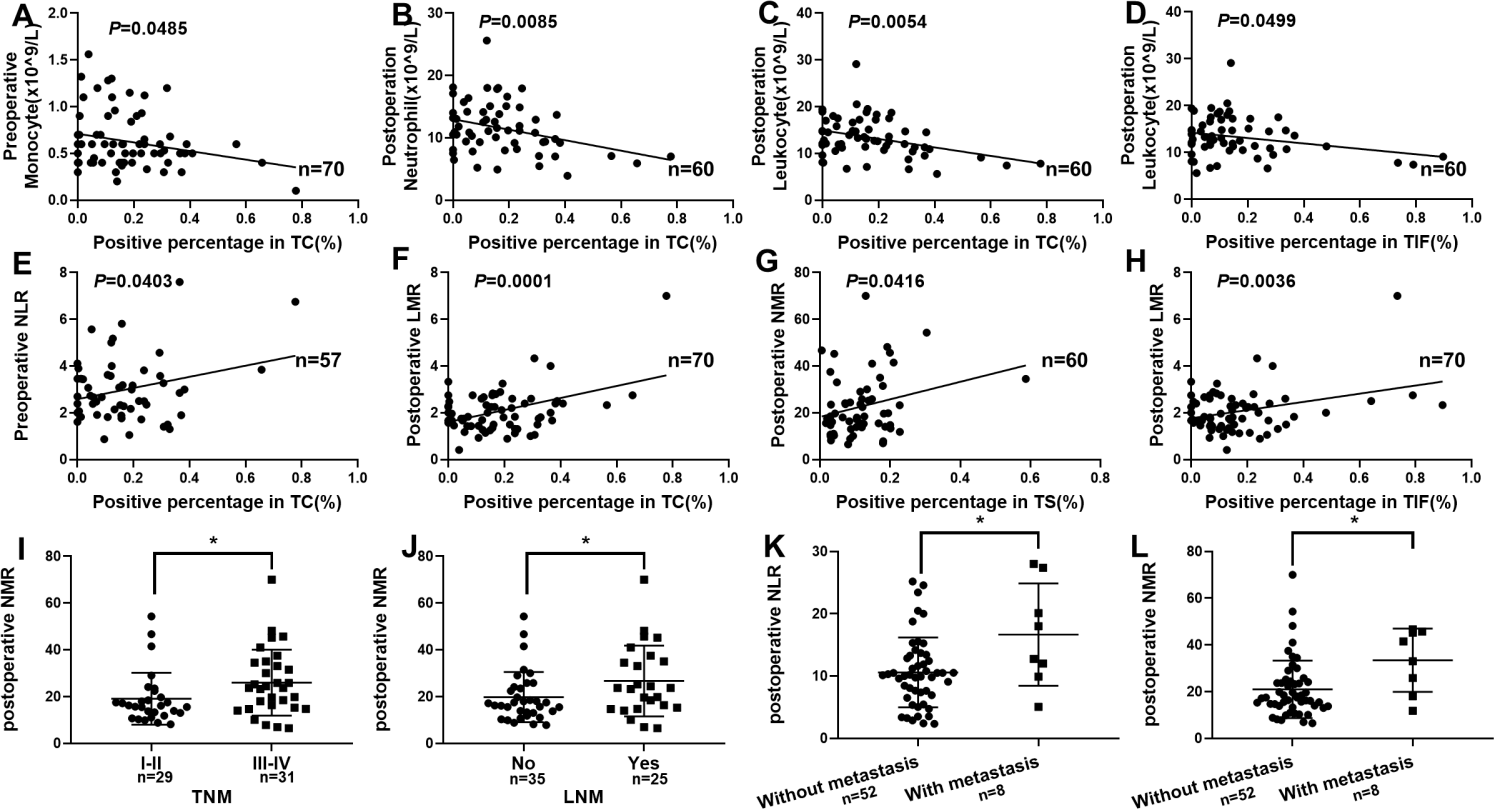
Correlation between CXCL17 and various peripheral blood indicators in OSCC. **(A–D)** CXCL17 expression was inversely associated with monocyte, neutrophil and leukocyte. **(E–H)** Correlation between CXCL17 of distinct regions and NLR, LMR, NMR, respectively. **(I–L)** Patients with higher postoperative NMR showed higher TNM stage, higher lymph node metastasis rate and postoperative distant metastasis. *P <0.05, statistically significant.

Chronic inflammation and infections are known to promote angiogenesis, contributing to cancer initiation and progression, which can be characterized by the component fluctuation of neutrophil, lymphocyte and monocyte. To determine the potential role of CXCL17 in immune cell regulation in OSCC, we performed comprehensive analyses of immune cell infiltration and examined the correlation between CXCL17 expression and neutrophil, lymphocyte and monocyte using complete blood count analysis. As it is shown, CXCL17 expression in TC was slightly inversely associated with preoperative monocyte (*P* = 0.0485, **Figure 5A**), but exhibited a strong inverse relationship with postoperative neutrophil (*P* = 0.0085, **Figure 5B**) and postoperative leukocyte (*P* = 0.0054, **Figure 5C**). Besides, CXCL17 expression in TIF was slightly negatively correlated with postoperative leukocyte levels (*P* = 0.0499, **Figure 5D**). These results indicated that OSCC patients with high CXCL17 expression in TC tend to recruit fewer neutrophil and leukocyte after surgery which may effected the prognosis of OSCC. Furthermore, CXCL17 in TC was significantly correlated with preoperative NLR (*P* = 0.0403, **Figure 5E**) and postoperative LMR (*P* = 0.0001, **Figure 5F**). Also, CXCL17 in TS was positively associated with postoperative NMR (*P* = 0.0416, **Figure 5G**). CXCL17 in TIF was strongly associated with postoperative LMR (*P* = 0.0036, **Figure 5H**). Moreover, we investigated the correlation of these indicators with clinicopathologic feature of OSCC patients. Patients with higher postoperative NMR showed higher TNM stage (*P* = 0.0397, **Figure 5I**) and a higher lymph node metastasis rate (*P* = 0.0432, **Figure 5J**). Additionally, patients with postoperative distant metastasis exhibited higher postoperative NLR (*P* = 0.0102, **Figure 5K**) and higher postoperative NMR (*P* = 0.0108, **Figure 5L**).However, the accuracy of the conclusion and the underlying mechanism warrant further investigation.

## 4 Discussion

The mucosal immune network serves as a crucial physical barrier that prevents pathogens from entering the body. Damage to the mucosal barrier can trigger inflammation, and chronic or recurrent inflammation is associated with the development of various human tumors, including gastric and oral cancers. The network of immune cells that mediates the defensive mechanisms in the mucosa is likely shaped by chemokines, which attract a wide range of immune cells to specific sites of the body. Chemokines are essential for recruiting various immune cells to the tumor microenvironment. CXCL17 is a mucosal-associated homeostatic chemokine that recently proved to be related to infection, inflammation, angiogenesis and tumorigenesis. However, its clinical significance in OSCC remains largely unexplored. Overexpression of CXCL17 has been observed in conditions such as idiopathic pulmonary fibrosis, gastrointestinal, breast and lung cancer, endometrial and pancreatic carcinoma (35). In intraductal papillary mucinous adenoma, CXCL17 was found to be upregulated exclusively during tumor development and thought to be involved in immune surveillance (14). In this study, we have innovatively found that CXCL17 expression is significantly elevated in OSCC tissues compared to normal mucosa, and the proportion of CXCL17^+^ cells increased throughout the process of malignant transformation. Consistent with GEPIA database analysis, CXCL17 RNA expression was also elevated in various other cancers, including cervical squamous cell carcinoma, endocervical adenocarcinoma, and lung adenocarcinoma. Furthermore, CXCL17 has been associated with decreased overall survival and disease-free survival in pancreatic adenocarcinoma. These findings prompted us to further investigate the clinical significance of CXCL17 in OSCC.

Immunohistochemical analysis confirmed that both tumor cells and stromal cells express CXCL17, with significantly higher expression in tumor cells than in stromal cells. Notably, no significant difference in CXCL17 expression was observed between TC and TIF for either tumor cells or stromal cells, indicating a lack of regional heterogeneity in CXCL17 distribution in OSCC. Moreover, we identified a strong correlations between CXCL17 expression and postoperative metastasis in TC, TS and TIF. Previous studies have revealed that breast cancer cells can secrete CXCL17, which increased the accumulation of metastatic CD11b^+^Gr-1^+^ myeloid-derived suppressor cells (MDSCs) in the lungs, and in turn, ultimately promote lung metastases of breast cancer (36). Analogously, CXCL17 has been implicated in modulating the LKB1-AMPK axis to reinforce malignant invasion of tumor cells and suppresses autophagy in hepatocellular carcinoma (37). However, the specific mechanism through which CXCL17 contributes to metastasis in OSCC remain poorly understood, warranting further investigation into this important area of research.

The prognosis value of CXCL17 in OSCC has not been extensively studied. Only a few investigations have noted CXCL17 expression in oral cancer (13) and Sjögren’s syndrome (38) without in-depth analysis. Our study, consistent with previous findings from the GEPIA analysis, demonstrated a significant correlation between CXCL17 expression and prognosis in OSCC patients. In tumor nest, high CXCL17 expression in both tumor cells and stroma cells was strongly associated with decreased overall survival and metastasis-free survival, although it did not correlate with recurrence-free survival or disease-free survival. Furthermore, in the invasive front, CXCL17^+^ tumor cells tended to be a risk factor for metastasis-free survival, while CXCL17^+^ stromal cells got no effect on OS, metastasis-free survival, recurrence-free survival or disease-free survival. Importantly, the Cox proportional hazards model revealed that tumor cell-derived CXCL17 in TC was an independent risk factor for overall survival rate in OSCC. These findings provide new evidence that CXCL17 was involved in malignant transformation of OSCC and suggest its potential utility as a valuable marker for assessing tumor behavior and patient prognosis in OSCC.

CXCL17 and its receptors play a crucial role in many pathophysiological processes, influencing not only the trafficking of leukocytes to tumors but also modulating the tumor immune response, which can ultimately lead to tumor metastasis (39). As a homeostatic chemokine, CXCL17 is a potent chemoattractant for monocytes, neutrophil (40), dendritic cells and macrophages (9, 41). Although CXCL17 expression is induced under inflammatory conditions, it may also exhibit anti-inflammatory properties. *In vitro* experiments on J774 cells or primary macrophages that were pretreated with 4-Cys mature CXCL17 and subsequently stimulated with lipopolysaccharide (LPS) showed a reduction in the expression of pro-inflammatory markers such as IL-6, TNF-α/β, and iNOS, compared to the macrophages treated with LPS alone (42). Collectively, these studies suggest that CXCL17 may play a dual role in the inflammatory response, contributing to both the promotion of immune cell recruitment and the modulation of inflammatory signaling.

The most commonly analyzed parameters in the literature are the absolute counts of neutrophils, monocytes, and lymphocytes, as well as the ratio between all these immunocytes such as NLR, NMR and LMR. In our study, higher CXCL17 expression in TC was strongly related with lower postoperative neutrophil counts, lower preoperative monocyte counts, and lower postoperative leukocyte counts. Additionally, CXCL17 in TIF showed an inverse relationship with postoperative lymphocyte counts. These findings suggest that CXCL17 may facilitate the migration of monocytes to the tumor site, resulting in decreased monocyte levels in peripheral blood. A meta-analysis of 5475 head and neck cancer patients found that elevated pretreatment NLR in peripheral blood was associated with poorer prognosis and increased likelihood of local invasion and distant metastasis (43). Furthermore, our study highlighted the significant correlations between CXCL17 and various blood parameters, including preoperative NLR and postoperative LMR. CXCL17^+^ stromal cells in tumor nest was positively associated with postoperative NMR. Patients with higher CXCL17^+^ tumor cells in tumor front had higher postoperative LMR. Many studies have reported that a high NLR is related to adverse pathological characteristics in various tumor (44). We further investigated the association between CXCL17 and clinicopathological features in OSCC patients. Patients with higher postoperative NMR showed higher TNM stage and higher lymph node metastasis rate. Consistent with other studies, we also found that patients with postoperative distant metastasis showed higher postoperative NLR and higher postoperative NMR. Overall, these findings underscore the importance of CXCL17 in modulating immune responses in OSCC and suggest that further investigation into its mechanisms could provide valuable insights into its role in recruiting peripheral blood cells and shaping the immune landscape in tumors.

Although our findings establish tumor cell-derived CXCL17 in the TC as a promising independent prognostic factor for OSCC, potentially through modulation of immune responses, this study has several limitations. First, its retrospective nature and single-center patient cohort may introduce selection bias and limit the generalizability of our conclusions. Second, the specific cellular sources of CXCL17 and its precise mechanistic role in OSCC pathogenesis remain incompletely elucidated. It is unclear whether it primarily functions in immune cell recruitment, angiogenesis, or other signaling pathways. Furthermore, the absence of *in vitro* or *in vivo* functional validation—such as chemotaxis assays or immune cell subset analysis—precludes causal inference regarding the relationship between CXCL17 and immune cells. We suggest that future mechanistic studies, including Transwell-based chemotaxis assays, neutralizing antibody-based animal models, single-cell RNA sequencing, or laser capture microdissection (LCM)-based qPCR, are warranted to elucidate the underlying biology. Lastly, the clinical applicability of CXCL17 as a standardized biomarker requires the development of a definitive scoring system and validation of a cost-effective, routine immunohistochemical assay.

In conclusion, our findings demonstrate that tumor cell-derived CXCL17 in the tumor core is a promising independent prognostic factor for OSCC, potentially through modulating immune responses. Future research will be directed along three paths. First, we will further expand the sample size to improve the accuracy of the research results and establish a clinically viable scoring cutoff. Second, we will employ *in vitro* and *in vivo* models to dissect the functional mechanisms of CXCL17, particularly its interaction with specific immune cell populations in the tumor microenvironment. Finally, given its independent prognostic value, CXCL17 expression in the tumor core may serve as a potential biomarker for risk stratification in OSCC patients. Further validation in prospective multicenter cohorts is warranted to confirm its clinical utility.

## 5 Conclusion

CXCL17 is closely related to angiogenesis, tumorigenesis, infection and inflammation, and is increasingly attracting people’s attention. Here, we explored the expression pattern of CXCL17 and revealed its correlation with tumor TNM stage and distant metastasis of OSCC. Judging from its clinical significance, we put a high value on the diagnostic value of CXCL17 in OSCC patients, aiding in more accurate diagnoses and treatment planning. Additionally, our findings regarding the interactions between CXCL17 and peripheral blood immune indexes further illuminate its role in modulating the immune response during tumor progression, reinforcing its importance in cancer biology. Overall, our research contributes valuable insights into the clinical significance of CXCL17 and supports further exploration of its therapeutic potential and implications in cancer management of OSCC.

Based on the findings of this study, the future clinical course should focus on translating our prognostic marker into practical applications for precision surgery and treatment. Specifically, we aim to develop a standardized molecular assay based on our signature along with other molecular indicators or clinical parameters to intraoperatively assess tumor margins, guiding the extent of resection to achieve a molecularly “negative” margin. This approach could directly inform adjuvant therapy decisions, allowing for more personalized and intensified treatment for high-risk patients, ultimately aiming to improve locoregional control and long-term survival outcomes.

## Data Availability

The original contributions presented in the study are included in the article/supplementary material. Further inquiries can be directed to the corresponding authors.
